# In Vitro and Field Effectiveness of the Combination of Four *Trichoderma* spp. Against *Sclerotinia sclerotiorum* and Its Impact on Potato (*Solanum tuberosum* L.) Crop Production

**DOI:** 10.3390/plants15010156

**Published:** 2026-01-04

**Authors:** Gabriel Herrera-Rodriguez, Ruben Felix-Gastelum, Maria Belen Irazoqui-Acosta, Sara Elodia Armenta-Lopez, Rosa Maria Longoria-Espinoza, Francisco Javier Orduño-Espinoza, Jessica Maria Parra-Parra

**Affiliations:** 1 Local Plant Health Board of the Fuerte Valley, Lazaro Cardenas, 315 Pte. Col. Centro, Los Mochis CP 81200, Sinaloa, Mexico; gabrielherrera44@hotmail.com (G.H.-R.); biobelen_irazoqui@hotmail.com (M.B.I.-A.); sararmenta@gmail.com (S.E.A.-L.); francisco_jvr@hotmail.com (F.J.O.-E.); jessicaparra115@gmail.com (J.M.P.-P.); 2 Los Mochis Unit, Department of Natural and Exact Sciences, Autonomous University of the West, Macario Gaxiola Boulevard and International Highway S/N, Los Mochis CP 81223, Sinaloa, Mexico; rosa.longoria@uadeo.mx

**Keywords:** antagonists, biological control, inhibition, metabolites

## Abstract

White mold (*Sclerotinia sclerotiorum*) reduces potato yield and quality in Sinaloa, Mexico. This study first evaluated the in vitro efficacy of *Trichoderma azevedoi*, *T. afroharzianum*, *T. asperellum* and *T. asperelloides* in inhibiting *S. sclerotiorum* mycelial growth and sclerotia production. Field experiments then assessed a combination of these antagonists, their alternating application with synthetic fungicides, and a fungicide-alone treatment for disease control, sclerotia reduction and yield increase. In vitro, all four *Trichoderma* species significantly inhibited the pathogen, achieving 60.1–63.1% mycelial suppression in dual culture and 90.3–94.1% via volatile metabolites, with the latter also completely suppressing sclerotia formation. In the field, the *Trichoderma* combination significantly controlled white mold, reducing plant incidence and severity to 66.0 and 27.1% in 2021 and 55.6 and 18.8% in 2022, while lowering sclerotia production to 32.7 and 14.6 on ten plants, respectively. This control extended to tubers, where incidence and severity were reduced to 1.6% and 0.4% in 2021, and 1.3% and 0.3% in 2022. The alternating application of *Trichoderma* with synthetic fungicides proved statistically equivalent to the *Trichoderma*-alone treatment in disease control, while the fungicides-alone treatment was significantly less effective. Potato yield was highest in plots treated with the *Trichoderma* combination (46.0 and 52.9 t ha^−1^ in 2021 and 2022, respectively). These results highlight the potential of using a mixture of these four *Trichoderma* species as a cornerstone of sustainable disease management in Sinaloa, offering effective control of potato white mold while significantly reducing dependence on synthetic fungicides.

## 1. Introduction

The potato is a globally essential food crop. In Mexico, its cultivation represents a multi-million-dollar industry and a significant component of agricultural production. However, fungal diseases are a primary limiting factor for potato yield and quality, with white mold (*Sclerotinia sclerotiorum*) being a particularly damaging pathogen [1,2].

Fungal diseases are a primary limiting factor for potato production and quality. Among the most significant are soft rot of tubers (*Sclerotium rolfsii*) and white mold (*Sclerotinia sclerotiorum*) [3,4].

White mold management is challenging because the pathogen affects more than 600 species of Dicotyledonae and has also been recorded in some monocotyledonous species [5,6,7]. It can behave as an endophyte in rice (*Oryza sativa*), wheat (*Triticum aestivum*), corn (*Zea mays*), barley (*Hordeum vulgare*) and oat (*Avena sativa*) [8]. Economic losses associated with white mold in the United States of America reach USD 200 million annually [9]. In Sinaloa, Mexico, it has been reported in eggplant and beans with up to 50% damage, while potato losses are estimated at around 30% [4,10,11].

The infectious cycle of *S. sclerotiorum* begins when soil-borne sclerotia germinate to produce hyphae that directly penetrate the stem, affecting leaves and fruits [12]. Alternatively, they can germinate carpogenically, forming apothecia that release ascospores. These spores require prolonged leaf wetness periods, temperatures of 15–20 °C and senescent tissues to establish infection [13]. Once inside the host, the fungus produces cellulase, pectinase and oxalic acid, which promote cell wall degradation, defense suppression and tissue necrosis [9,14,15]. Subsequently, colonized tissues form a mass of hyphae that gives rise to sclerotia: compact, melanized, black structures capable of remaining viable for up to 10 years, depending on environmental conditions [16,17].

Due to the low resistance levels in host crops, various chemical compounds are used for controlling white mold. In the United States, Canada, Australia, China and European countries, control strategies for white mold commonly rely on fungicides with different modes of action. These include succinate dehydrogenase inhibitors such as boscalid, fluxapyroxad, and penthiopyrad; inhibitors of mitochondrial respiration like pyraclostrobin, picoxystrobin, and trifloxystrobin; and demethylation inhibitors including prothioconazole and tetraconazole. Additionally, microtubule assembly inhibitors such as thiophanate-methyl and oxidative phosphorylation uncouplers like fluazinam are frequently integrated into management programs across major potato-producing regions [18,19,20]. In Sinaloa, the same groups of fungicides are applied for controlling white mold disease, achieving 85–90% control in preventive applications [11]. However, this strategy has become increasingly ineffective as the fungus has developed resistance to synthetic fungicides, leading to a cycle of increased application rates and environmental contamination [21]. These challenges highlight the critical need for sustainable alternatives, such as biological control, to manage white mold effectively [21].

Different strains of *Trichoderma* have been used to manage white mold with promising results. In Brazil, *T. asperelloides* reduced the development of white mold in soybean [22]. In Egypt, *T. harzianum* and *T. viride* limited the pathogen’s impact on dry bean [23]. In India, *T. erinaceum* and *T. viride* suppressed stem rot caused by *S. sclerotiorum* in common bean, and *T. afroharzianum* and *T. lixii* were successfully used for the biological control in mustard [24,25]. In Turkey, *T. harzianum* inhibited the disease in lettuce plants [26].

*Trichoderma* species suppress *S. sclerotiorum* through multiple mechanisms, including competition for space and nutrients, antibiosis via metabolite production, and mycoparasitism directly parasitizing the pathogen’s mycelium, sclerotia, and apothecia [27,28]. This reduction in primary inoculum helps decrease disease incidence in subsequent growing seasons [29].

In Mexico, there are no in vitro or field studies focused on managing potato white mold using antagonistic fungi [4]. Therefore, this work aimed to: (a) evaluate the effectiveness of four *Trichoderma* species against *S. sclerotiorum* both in vitro and under field conditions; (b) determine the efficacy of alternating applications of these antagonists with synthetic fungicides; (c) compare these treatments with fungicides applied alone and (d) assess their impact on potato tuber yield in semi-commercial fields.

## 2. Results

### 2.1. Molecular Identification of Sclerotinia sclerotiorum

**Figure 1:**
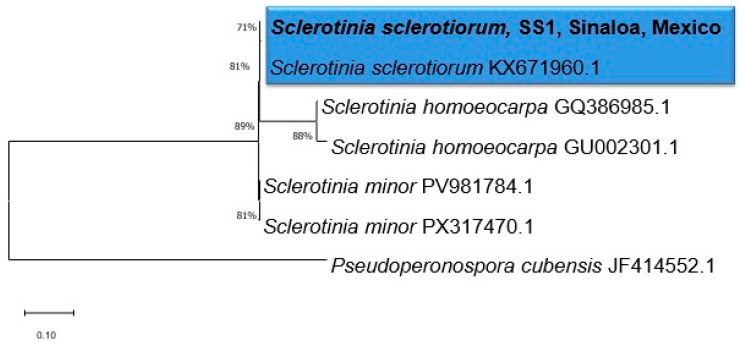
Phylogenetic analysis of the *Sclerotinia sclerotiorum* isolate from Sinaloa, Mexico. The maximum likelihood tree was constructed based on the internal transcribed spacer (ITS) region. The sequence of *Pseudoperonospora cubensis* (JF414552.1) was used as an outgroup. The isolate is shown in bold. Bootstrap values greater than 60% are shown at the nodes.

The maximum-likelihood tree generated with the ITS region sequences showed that the isolate SS1 (GenBank accession PX471991.1) from Sinaloa, Mexico, clusters with the *S. sclerotiorum* with reference sequence (KX671960.1) and high bootstrap support (71%), confirming its identity as *S. sclerotiorum* (Figure 1).

### 2.2. In Vitro Inhibition of Sclerotinia sclerotiorum by Four Trichoderma Species

The in vitro antagonism assays revealed significant differences in the inhibition of *S. sclerotiorum* mycelial growth by the four *Trichoderma* species (DF = 3; *F* = 19.38; 28 *p* < 0.0001). In dual cultures, the inhibition percentages ranged from 60.1 to 63.1% (Table 1). *Trichoderma asperellum* showed the highest inhibition at 63.1%; standard deviation (SD) 0.98 (class 3), followed by *T. afroharzianum* (62.8%; SD 0.59, class 3), *T. asperelloides* (61.8%; SD 0.83, class 3) and *T. azevedoi* (60.1%; SD 0.93, class 3) (Table 1). These differences, while statistically significant, represent a narrow biological range of just 3% in actual growth inhibition.

Sclerotia production by *S. sclerotiorum* in dual cultures varied significantly among treatments (DF = 4; *F* = 555.18; *p* < 0.0001). The lowest numbers of sclerotia per Petri dish were observed in confrontations with *T. asperelloides*, *T. azevedoi* (both 4.0) and *T. afroharzianum* (4.3; SD 0.89), with no significant differences among them. In contrast, the interaction with *T. asperellum* resulted in 6.8, SD 0.89, sclerotia per dish. All treatments significantly reduced sclerotia compared to the control, with *T. asperelloides* and *T. azevedoi* being the most effective (Table 1). This represents a strong biological effect, with a reduction of up to 16.8 sclerotia compared to the control.

The volatile metabolites produced by the four *Trichoderma* species also significantly inhibited the pathogen, reducing mycelial growth by 90.3, SD, 1.94 to 94.1%, SD 2.89, with no significant differences among the species (DF = 3; *F* = 3.8; *p* < 0.0001), with *T. afroharzianum* showing the highest mycelial growth reduction (94.1%; SD 2.89), followed by *T. asperellum* (93.5%; SD 1.62), *T. asperelloides* (91.0%; SD 3.05) and *T. azevedoi* (90.3%; SD 1.94), though no significant differences were observed among species (DF = 3; *F* = 3.8; *p* < 0.0001). Although statistically significant, the 3.8% difference in mycelial growth inhibition among species represents a narrow biological range. In sclerotia production, all *Trichoderma* species demonstrated complete suppression, showing no significant differences among them in this parameter.

### 2.3. Hyphal Interactions Between Four Trichoderma spp. and Sclerotinia sclerotiorum

**Figure 2:**
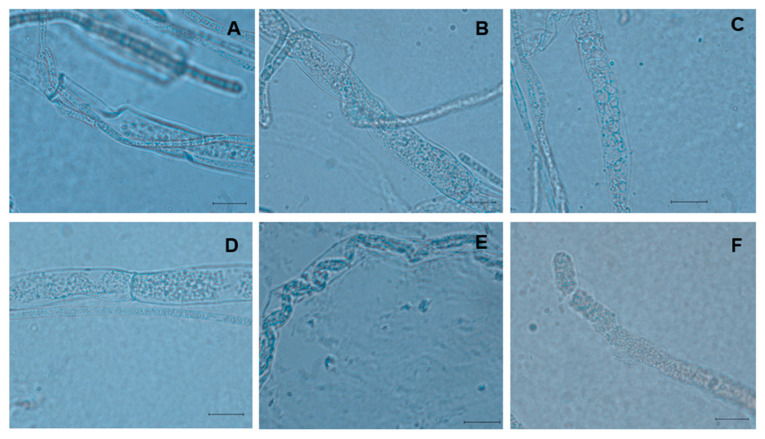
Hyphal interactions of four *Trichoderma* spp. with *S*. *sclerotiorum*. (**A**) Adhesion *T. asperellum* TAM74, (**B**) coiling *T. asperelloides* TES24, (**C**) granulation *T. afroharzianum* TAF75, (**D**) vacuolation *T. asperellum* TAM74, (**E**) penetration *T. asperellum* TAM74 (**F**) lysis *T. asperellum* TAM74. Scale = 10 µm.

The four *Trichoderma* species exhibited a range of hyphal interactions with *S. sclerotiorum*, including adhesion, coiling, granulation, vacuolization, penetration, and lysis (Figure 2A–F). All species showed common mechanisms of adhesion, coiling, and granulation. *T. asperellum* displayed the most complex activity, inducing adhesion, vacuolization, direct penetration and subsequent lysis of the pathogen’s hyphae. *T. afroharzianum* also demonstrated vacuolization and penetration. In contrast, *T. asperelloides* was only observed to induce vacuolization, while *T. azevedoi* exhibited penetration followed by lysis (Table 2).

### 2.4. Efficacy of Trichoderma spp., Synthetic Fungicides and Their Alternate Application for Controlling Potato White Mold Under Field Conditions

In the 2021 trial, the lowest disease incidence in semi-commercial plots was observed in treatments with the *Trichoderma* spp. combination or its alternate application with synthetic fungicides. No significant differences were found between these two treatments, but both differed significantly from the plots treated with synthetic fungicides alone (DF = 2; *F* = 13.64; *p* < 0.0001; Table 3). Disease severity in plants ranged from 27.1, SD 7.26, to 61.7%, SD 8.68, with significant differences among treatments (DF = 2; *F* = 2.75; *p* < 0.0001). The combination of the four *Trichoderma* species provided the best disease control (Table 3).

In 2022, disease incidence varied from 55.6, SD 7.26, to 75.6%, SD 8.68, showing significant differences among treatments (DF = 2; *F* = 5.13¸ *p* = 0.0140). The lowest incidence was recorded in plots treated with the *Trichoderma* combination or the alternate application with fungicides, with no significant differences between them (DF = 2; *F* = 5.07; *p* = 0.0146), but both were significantly lower than the fungicide-alone treatment (Table 3). Disease severity ranged from 18.8, SD 5.42, to 33.8%, SD 6.31, differing significantly among treatments (DF = 2; *F* = 2.75; *p* < 0.0001), with the *Trichoderma* combination again providing the best control (Table 3).

### 2.5. Effectiveness of Four Trichoderma Species, Synthetic Fungicides and Their Alternate Use on Sclerotia Production by Sclerotinia sclerotiorum in Potato Plants Under Field Conditions

In 2021, the production of sclerotia by *S. sclerotiorum* per treatment ranged from 32.7, SD 2.01, to 167.7, SD 3.14. The lowest number of sclerotia was recorded in plants treated with the *Trichoderma* spp. combination, followed by the alternate application of *Trichoderma* spp. with synthetic fungicides treatment. No significant differences were observed between these two treatments (DF = 2; *F* = 18.6; *p* < 0.0001), but both resulted in significantly fewer sclerotia than the treatment with synthetic fungicides alone (Table 4).

In 2022, the lowest sclerotia production was also observed in plants treated with the *Trichoderma* mixture or the alternate application with fungicides. No significant differences were found between these treatments (DF = 2; *F* = 13.91; *p* < 0.0001), but both were significantly more effective than the fungicides-alone treatment, which yielded a significantly higher average of 93.8, SD 2.88, sclerotia per treatment (Table 4).

### 2.6. Effectiveness of Four Trichoderma spp., Synthetic Fungicides and Their Alternate Use in Controlling White Mold on Potato Tubers Under Field Conditions

Tuber disease incidence and severity were consistently lowest in the *Trichoderma* combination and the alternate application treatments across both years (Table 5). Statistically, these two treatments showed no significant difference from each other in either incidence (2021: *F* = 3.95, *p* = 0.0328; 2022: *F* = 6.84, *p* = 0.0045) or severity (2021: *F* = 1.83, *p* = 0.1360; 2022: *F* = 1.22, *p* = 0.3479). Biologically, this demonstrates their equivalent efficacy in controlling tuber infection.

In contrast, both biological treatments provided significantly superior control compared to the synthetic fungicides applied alone. For disease incidence in 2021, the *Trichoderma* combination (1.6%; SD 4.75) and alternate application (4.5%; SD 6.22) were significantly lower than the fungicide-only treatment (7.4%; SD 7.78). This pattern was confirmed in 2022, with incidences of 1.3%, SD 0.87, and 3.3%, SD 2.77, for the biological treatments, respectively, compared to 6.2%, SD 4.42, for fungicides alone. A similar significant advantage was observed for disease severity in both years (Table 5).

### 2.7. Production of Tubers in Plots Sprayed with a Combination of Four Trichoderma spp., Synthetic Fungicides or the Alternate Use of These Treatments

Potato tuber yield in 2021 ranged from 42.4, SD 2.15, to 46.0, SD 3.13 t ha^−1^, showing significant differences among treatments (DF = 2; *F* = 4.06; *p* = 0.0303). The highest yield was obtained in plots treated with the *Trichoderma* combination, while the lowest yield was recorded in plots treated with synthetic fungicides alone (Table 6).

In 2022, yields were higher, ranging from 44.5, SD 7.87, to 52.9, SD 5.04 t ha^−1^, with significant differences among treatments (DF = 2; *F* = 4.09; *p* = 0.0295). Plots treated with synthetic fungicides alone showed the lowest yield (44.5; SD 7.87 t ha^−1^), which was significantly lower than the yield in plots treated with the *Trichoderma* combination (52.9; SD 5.04 t ha^−1^) (Table 6).

## 3. Discussion

The molecular analysis confirmed the isolate from symptomatic potatoes in Sinaloa is *S. sclerotiorum*, which is consistent with those reported by Kurt et al. [31]. This precise identification is crucial, given the limitations of morphological methods for this species [32,33]. This study provides a molecularly confirmed local isolate of *S. sclerotiorum* from the potato production system of Sinaloa, Mexico. The use of this genetically identified, region-specific isolate in all subsequent biocontrol assays ensures that the results are directly relevant to the local agricultural context.

In the in vitro tests, the four *Trichoderma* species significantly inhibited the mycelial growth of this local *S. sclerotiorum* isolate. This inhibitory effect aligns with reports of various *Trichoderma* species against the pathogen worldwide [25,28,34,35,36,37,38]. Furthermore, the production of sclerotia by *S. sclerotiorum* was strongly suppressed in dual cultures, a finding consistent with other studies [28]. The in vitro suppression of sclerotia formation in dual cultures (Table 1) is attributed primarily to resource competition, supported by the observed mycoparasitic colonization of sclerotia by *Trichoderma* spp. This combined activity correlates with the field results, demonstrating reduced disease pressure and offering a viable pathway for sustainable long-term management of white mold.

In our study, volatile metabolites from all four *Trichoderma* species significantly inhibited *S. sclerotiorum* mycelial growth (90.3–94.1%) and completely suppressed sclerotia production (Table 1). These results align with previous reports of *Trichoderma*-mediated inhibition through volatiles [25,27,28,37], and specifically confirm the complete suppression of sclerotia formation, a phenomenon also observed by Sridharan et al. [39]. Among the volatile compounds reported to have such bioactivity in other *Trichoderma* strains, alcohols and ketones, such as 1-Octen-3-one, 1-Octen-3-ol, Phenylethanol, and Carvomenthone from *T*. *azevedoi,* have been shown to inhibit both mycelial growth and sclerotia formation [40]. While the exact identity and mode of action of these volatile compounds remain unknown, our findings open new research avenues to characterize the specific metabolites produced by these *Trichoderma* strains and elucidate their mechanisms of action.

The *Trichoderma* species in this study exhibited the same hyphal interactions previously reported in other studies, including adhesion, coiling, granulation, penetration, and lysis [25,35,36]. These interactions, observed under direct contact conditions, are mediated by the production of hydrolytic enzymes. According to Ojaghian et al. [41], the endochitinases Chit33 and Chit37, as well as the glucanases A13gluc (α-1,3-glucanase) and B16gluc (β-1,6-glucanase) from *T. viridescens*, contribute to the degrade of the cell wall of *S. sclerotiorum*; whereas in the degradation of sclerotia, the endochitinases (Chit33 and Chit37) and a proteinase (Prb1), produced by *T. asperellum*, are also involved [42]. These observed mechanisms support their efficacy and likely contributed to successful field control. Future research should identify enzymes and specific volatile and non-volatile metabolites involved and their modes of action to optimize white mold management in Sinaloa, Mexico, for potatoes and other regional crops.

The combination of *T. azevedoi*, *T. afroharzianum*, *T. asperellum* and *T. asperelloides* was the most effective treatment for managing white mold in potato plants and tubers, outperforming synthetic fungicides. These results agree with Iqbal-Faruk [43], who found combined applications of *Trichoderma*, *Bacillus* and amendments effective in mustard, bean and pea. Similarly, Zeng et al. [44] reported reduced disease severity in soybeans with *T. harzianum* and Geraldine et al. [45] observed reduced apothecia production and disease severity in beans with *T. asperellum*.

The combination of *T. azevedoi, T. afroharzianum, T. asperellum*, and *T. asperelloides* was the most effective treatment for managing white mold in potato plants and tubers under Sinaloa field conditions, consistently outperforming synthetic fungicides. This study provides direct evidence of its efficacy in a key Mexican potato-producing region. Our results align with previous work demonstrating the utility of combined *Trichoderma* applications [43] and confirm their ability to significantly reduce sclerotia production, a crucial outcome for sustainable long-term control, as *Trichoderma* spp. act as mycoparasites on these survival structures [46,47].

Plots treated with the *Trichoderma* combination or its alternation with fungicides yielded the highest production in our Sinaloa field trials. This yield improvement is attributed to effective disease control and extended crop longevity, mediated by mechanisms including competition, mycoparasitism, antibiosis, and induced systemic resistance [48,49]. Our results are consistent with other regional studies reporting yield increases with *Trichoderma* applications [50,51], while certain fungicides provided disease control, they did not result in a corresponding yield increase [52]. These findings confirm the dual role of this *Trichoderma* combination in suppressing disease and enhancing yield, outperforming synthetic fungicides alone. The efficacy demonstrated in semi-commercial plots supports scaling this strategy to commercial levels in Sinaloa, reducing reliance on synthetic fungicides. Future research should evaluate these antagonists against other potato phytopathogens to develop more sustainable and cost-effective integrated management strategies.

Thirty-one thousand hectares of potatoes are cultivated in Sinaloa and Sonora, Mexico, where growers have shown interest in adopting this biological approach for disease management. Currently, the four endemic *Trichoderma* species are applied across 1500 hectares for both fresh-market and industrial potato production, with satisfactory results in controlling white mold. This successful implementation could serve as a starting point for broader adoption by other growers in the region and in potato-producing zones across Mexico.

The *Trichoderma* strains are produced in the growers’ own on-site laboratories, resulting in a more cost-effective strategy than synthetic fungicides; furthermore, growers have modern equipment for applying *Trichoderma* species at the time of planting and subsequent treatments by sprinkler irrigation systems, facilitating efficient and scalable implementation.

## 4. Materials and Methods

### 4.1. Obtaining Trichoderma Isolates and Molecular Identification of Sclerotinia sclerotiorum

This study utilized four *Trichoderma* isolates, *T. asperellum* (TAM74; OR521164), *T. asperelloides* (TES24; OR521181), *T. afroharzianum* (TAF75; OR521183), and *T. azevedoi* (TAI73; OR521182), selected from 26 isolates obtained from potato-growing regions in Sonora and Sinaloa, Mexico (Table 7), based on their superior performance in preliminary dual confrontation assays against *S. sclerotiorum*, showing the strongest mycelial growth inhibition. These isolates were provided by the Local Plant Health Board of the Fuerte Valley, Los Mochis, Sinaloa, Mexico [53]. The *S. sclerotiorum* (PX471991.1) isolate was obtained from symptomatic potato plants in northern Sinaloa. Symptomatic tissue was surface-disinfected with 1.0% sodium hypochlorite for one minute, followed by three rinses in sterile distilled water, and dried on sterile absorbent paper under aseptic conditions. Tissue fragments were then plated on PDA and incubated at 25 ± 2 °C. Pure cultures were obtained by transferring hyphal tips to fresh PDA medium. For long-term preservation, working cultures were maintained on Potato Dextrose Agar (PDA) slants at 4 °C, and master stocks were stored in 20% glycerol at 7 °C.

Prior to field application, the viability and concentration of each *Trichoderma* isolate were confirmed. Conidial suspensions were prepared from *Trichoderma* spp. cultures grown on ground corncob [54] for 15 days at 25 °C under a 12/12 h light/dark regimen. The concentration of colony-forming units (CFUs) was determined by serial dilution plating on PDA and incubation at 25 °C for 3 days. The final applied suspension was adjusted to a concentration of 1.6 × 10^7^ CFU mL^−1^ for each isolate, which was verified again immediately before treatment application.

For molecular identification, one 3 mm disk of the isolate was placed in 25 mL of nutrient broth (Becton Dickinson, Sparks, MD, USA). The culture was incubated at 27 ± 2 °C for five days with continuous agitation at 150 rpm (Labnet International, Inc.^®^, Edison, NJ, USA); afterwards, the mycelium was placed in 2 mL Eppendorf microtubes. Genomic DNA extraction was performed using the 2% CTAB protocol according to Sanger et al. [55]; the final DNA concentration was adjusted to 50 ng/µL. The purity and concentration of DNA were determined using a NanoDrop spectrophotometer (Thermo Fisher Scientific^®^, Madison, WI, USA).

The identification of *Sclerotinia sclerotiorum* isolate was carried out by polymerase chain reaction (PCR), targeting the amplification of an approximately 650 bp fragment corresponding to the Internal Transcribed Spacer (ITS) using the primers ITS1 (5′-TCC GTA GGT GAA CCT TGC GG-3′) and ITS4 (5′-TCC TCC GCT TAT TGA TAT GC-3′), described by White et al. [56]. 

The PCR reaction contained 2 µL of DNA (25 ng/µL), 1x of reaction buffer, 1.5 mM of MgCl_2_, 0.5 µM of each oligonucleotide, 0.2 µM dNTPs, and 0.5 U of Taq polymerase (Invitrogen^®^ Carlsbad, CA, USA) in a total volume of 25 µL. The PCR amplification was performed as follows: one cycle of 95 °C for 5 min, followed by 35 cycles of 95 °C for 45 s, 58 °C for 45 s and 72 °C for 45 s, and a final extension at 72 °C for 5 min. Amplifications were performed in a thermal cycler (BIORAD^®^; C1000 Thermal Cycler CFX96, Singapore). The amplified product was sent to Macrogen^®^ (Seoul, Republic of Korea) for sequencing.

Internal Transcribed Spacer (ITS) region was sequenced bidirectionally. Sequence editing and curation were performed using BioEdit version 7.2.5 [57], which included trimming regions with a Q-score below 30 to ensure high-quality data. The final consensus sequence was generated from the alignment of the forward and reverse reads, enabling verification of each nucleotide position for subsequent BLAST and phylogenetic analyses. The sequence was compared using the NCBI blastn web service (https://blast.ncbi.nlm.nih.gov/Blast.cgi?PAGE_TYPE=BlastSearch) (accessed on 14 October 2025). The sequence was aligned with reference sequences belonging to type strains of the different species within the *Sclerotinia* complex: *S. sclerotiorum* (KX671960.1), *S. minor* (PV981784.1, PX317470.1), and *S. homoeocarpa* (GQ386985.1, GU002301.1), using the MUSCLE software [58] implemented in MEGA X version 10.2.4 [59]. The sequence of *P. cubensis* (JF414552.1) was used as an outgroup in the phylogenetic analysis. Phylogenetic inference was performed using the Maximum Likelihood (ML) method in the program, applying the Tamura-Nei [60]. In the ML analysis, 1000 bootstrap replications were used. The phylograms were edited in FigTree v1.4.0 [61].

### 4.2. In Vitro Antagonism of Trichoderma spp. Against Sclerotinia sclerotiorum and Sclerotia Formation

The in vitro antagonistic effect of *T. asperellum*, *T. asperelloides*, *T. afroharzianum* and *T. azevedoi* against *S. sclerotiorum* was evaluated using the dual culture technique on Potato Dextrose Agar (PDA; BD, Becton Dickinson of Mexico). Mycelial discs (5 mm) from 3-day-old *Trichoderma* and 7-day-old *S. sclerotiorum* cultures were placed at opposite ends of 90 mm Petri dishes. The treatments were arranged in a completely randomized design with four replicates (four Petri dishes) per combination and were conducted twice. Controls consisted of individual cultures of each fungus under identical conditions. Plates were sealed with Parafilm and incubated in a growth chamber (BIORAD^®^; C1000 Thermal Cycler CFX96, ejemplo Singapore) under a photoperiod of 12 h light/12 h darkness at 25 ± 1 °C. Mycelial growth was measured every 24 h until day 4, when it was observed that the control plates were completely colonized. The inhibition percentage was calculated as I = [(C − T)/C] × 100, where C is the radial growth of *S. sclerotiorum* alone and T is its growth in confrontation with *Trichoderma* spp. [62]. For the final analysis, data from both independent trials were combined, providing a total of eight replicates per treatment.

Antagonistic efficacy was classified using Bell et al. [63] scale: Class 1 = *Trichoderma* covered the entire medium; Class 2 = *Trichoderma* overgrew at least two-thirds of the medium surface; Class 3 = both organisms colonized approximately one-half of the medium surface of the medium (more than one-third and less than two-thirds); Class 4 = *S. sclerotiorum* colonized at least two-thirds of the medium surface and appeared to withstand encroachment by *Trichoderma*; Class 5 = *S. sclerotiorum* covered the entire medium. Sclerotia production was quantified 15 days post-inoculation. The experiment was repeated once.

### 4.3. Effect of Volatile Metabolites from Trichoderma spp. on Mycelial Growth and Sclerotia Formation

The effect of volatile compounds was evaluated using the double plate method [64]. *Trichoderma* spp. and *S. sclerotiorum* were initially cultured on PDA for 3 days at 25 ± 2 °C. A 5 mm mycelial disc of *Trichoderma* spp. was placed in the center of a PDA plate and after 24 h, a disc of *S. sclerotiorum* was placed in another plate. The bases of both plates were coupled and sealed with Parafilm to create a shared gaseous environment without physical contact, with *Trichoderma* in the lower base and the pathogen in the upper one. The design was completely randomized with four replicates (four Petri dishes) and two independent trials were conducted. The coupled plates were incubated for 2 days in a growth chamber under a photoperiod of 12 h light/12 h darkness at 25 ± 1 °C. Controls included individual cultures of each organism. Antifungal activity was assessed when *S. sclerotiorum* fully colonized the control plates. Growth inhibition was calculated as described previously and sclerotia formation was evaluated 15 days after exposure. Data from the two independent trials were pooled for statistical analysis, yielding eight replicates per treatment.

### 4.4. Field Experiments

Experiments were conducted in a 15-ha commercial field with clay-loam soil (pH 7.2) naturally infested with *S. sclerotiorum* (0.2 sclerotia/kg soil, based on 27 samples). Trials were established on December 5, 2021, and November 18, 2022, using sprouted tubers of variety FL2027 (PepsiCo Mexico City, Mexico). Crop management followed Santos et al. [65]. Three treatments consisted of (a) biological: combination of *T. azevedoi*, *T. afroharzianum*, *T. asperellum* and *T. asperelloides* applied at planting (5.0 L ha^−1^, 1.6 × 10^7^ CFU mL^−1^) followed by five foliar applications (2.5 L ha^−1^) at 16-day intervals during the growing season; (b) chemical: Fluazinam (0.5 L ha^−1^, 250 g a.i. ha^−1^) applied twice at flowering onset and tolclofos-methyl (3 L ha^−1^, 2250 g a.i. ha^−1^) applied during tuberization and 15 days later; (c) alternation: alternating applications of *Trichoderma* spp. and synthetic fungicides at the described intervals and doses. The three treatments were arranged in a randomized complete block design with nine replications of 5558 m^2^ each. To avoid contamination from adjacent treatments, in the two central rows of each replication 18 m^2^ were selected as sampling points. Plant health was assessed one week prior to harvest, whereas tuber health and yield were evaluated at harvest time.

### 4.5. Disease Incidence, Severity and Sclerotia Production in Plants

Disease incidence was assessed at nine replicates per treatment (10 plants each). Severity was rated on a 0–5 scale (0 = no symptoms; 1 = 1–10%; 2 = 11–20%; 3 = 21–40%; 4 = 41–59%; 5 = 60–100% affected tissue) and calculated using the Townsend and Heuberger [30] formula: Severity = (∑(ni × vi)/(N × V)) × 100, where ni = category value, vi = number of plants in the category, V = highest category value (5) and N = total plants. Sclerotia were counted on 10 plants per sampling point.

### 4.6. Tuber Disease Evaluation and Yield

Tuber incidence and severity were assessed from nine replicates per treatment (two central rows, 18 m^2^ per point). Severity was rated on a 0–5 scale based on affected surface area and calculated using the Townsend and Heuberger [30] formula. Yield was determined by weighing harvested tubers from each sampling point.

### 4.7. Statistical Analysis

The data from each experiment were initially evaluated to test for normality (Shapiro–Wilk test) and homogeneity of variances (Bartlett’s test). The data from dual confrontation (mycelial growth inhibition and sclerotia formation) and from white mold incidence in tubers in 2022 did not meet these assumptions; therefore, they were analyzed using the non-parametric Kruskal–Wallis [66] test, and means were compared with the Conover [67] test using the InfoStat 2019e statistical package. Data on the in vitro effect of volatile metabolites on mycelial growth inhibition and sclerotia formation, as well as field data on sclerotia production, disease incidence and severity in plants including incidence and severity in 2021 and tuber yield under field conditions were analyzed by analysis of variance (ANOVA) using the SAS 9.0 package (SAS Institute Inc., Cary, NC, USA). Means were separated with Tukey’s test (*p* ≤ 0.05), following the methodology described by Little and Hills [68].

## 5. Conclusions

The combination of *T. afroharzianum, T. asperellum, T. asperelloides*, and *T. azevedoi* demonstrated efficacy in inhibiting *S. sclerotiorum* under both in vitro and field conditions. This treatment was associated with a significant reduction in the incidence and severity of white mold, correlated with decreased sclerotia production in potato plants and a corresponding reduction in tuber disease, alongside an increase in overall crop yield. The alternation of these antagonists with synthetic fungicides provided improved disease control compared to the exclusive use of fungicides. The field efficacy of these *Trichoderma*-based treatments indicates a viable path to reduce synthetic fungicide use. These results provide information on the potential of combined *Trichoderma* species applications as an effective and environmentally safe strategy for managing white mold in potato cultivation in Sinaloa, Mexico.

While this study demonstrates the successful short-term integration of *Trichoderma* species for white mold management, future research should address long-term aspects, including the sustained effects on soil health and microbial communities, the durability of plant resistance across multiple growing seasons, and monitoring potential development of resistance in *S*. *sclerotiorum* populations. Such a comprehensive assessment would strengthen the development of resilient integrated management strategies for potato production systems.

## Figures and Tables

**Table 1:** In vitro inhibition of *Sclerotinia sclerotiorum* mycelial growth and sclerotia production by four *Trichoderma* species using dual culture and volatile metabolites.

Dual Confrontation				Volatile Metabolites	
*Trichoderma* Isolate	% Inhibition	Scale	Number of Sclerotia	% Inhibition	Number of Sclerotia
*T. asperellum*	63.1 ± 0.98 a ^y^	3	6.8 ± 0.89 b ^y^	93.5 ± 1.62 a *	0.0 ± 0.00 b *
*T. afroharzianum*	62.8 ± 0.59 ab	3	4.3 ± 0.89 c	94.1 ± 2.89 a	0.0 ± 0.00 b
*T. asperelloides*	61.8 ± 0.83 b	3	4.0 ± 0.76 c	91.0 ± 3.05 a	0.0 ± 0.00 b
*T*. *azevedoi*	60.1 ± 0.93 c	3	4.0 ± 0.93 c	90.3 ± 1.94 a	0.0 ± 0.00 b
Control	^X^ NA	NA	20.8 ± 0.89 a	NA	27.0 ± 0.05 a
CV (%)	2.28		83.24	2.65	3.98

^X^ NA: Not applicable (no *Trichoderma* applied). ^y^ The data were analyzed using the nonparametric Kruskal–Wallis (*p* ≤ 0.05), statistical procedure, and mean separation was performed using the Conover procedure. * Means of eight replications followed by the same letter within a column are not significantly different according to Tukey’s test (*p* = 0.05).

**Table 2:** In vitro hyphal interaction of four *Trichoderma* species and *Sclerotinia sclerotiorum.*

Types of Hyphal Interactions ^x^						
*Trichoderma* Species	Adhesion	Coiling	Granulation	Vacuolization	Penetration	Lysis
*T. asperellum*	X ^y^	X	X	X	X	X
*T. afroharzianum*	X	X	X	X	X	
*T. azevedoi*	X	X	X		X	X
*T. asperelloides*	X	X	X	X		

^x^ The morphological changes and direct physical contact that occurred during hyphal interactions were determined using lactophenol-blue to stain the interaction zone of the fungi. ^y^ Positive interaction

**Table 3:** Effectiveness of four *Trichoderma* species, synthetic fungicides and their alternated use on the incidence and severity of white mold in potato plants under field conditions.

Treatment	Experiment 2021		Experiment 2022	
	Incidence (%)	Severity (%) ^x^	Incidence (%)	Severity (%)
*Trichoderma* spp. ^y^	66.0 ± 5.38 b *	27.1 ± 7.26 b	55.6 ± 7.26 b	18.8 ± 5.42 b
*Trichoderma* spp. alternating with synthetic fungicides ^z^	81.1 ± 12.31 ab	35.6 ± 9.94 ab	62.2 ± 9.94 b	27.0 ± 7.96 b
Synthetic fungicides	93.3 ± 12.20 a	61.7 ± 8.68 a	75.6 ± 8.68 a	33.8± 6.31 a
CV (%)	17.47	13.45	12.42	18.56

^x^ The severity of the disease was determined using the Townsend and Heuberger [30] formula. ^y^ *Trichoderma* spp.: *T. azevedoi*, *T. afroharzianum*, *T. asperellum* and *T. asperelloides*. ^z^ Synthetic fungicides: Mancozeb 5 L ha ^−1^, fluazinam 0.5 L ha ^−1^ and tolclofos methyl 3 L ha ^−1^. * Means of nine replications with a common letter across columns are not significantly different (*p* = 0.05, Tukey).

**Table 4:** Effectiveness of four *Trichoderma* species, synthetic fungicides and their alternate application on sclerotia production by *Sclerotinia sclerotiorum* in potato under field conditions.

Treatment	Sclerotia on 10 Plants	
	Experiment 2021	Experiment 2022
*Trichoderma* spp. ^y^	32.7 ± 2.01 b *	14.6 ± 2.31 b
*Trichoderma* spp. alternating with synthetic fungicides ^z^	40.8 ± 3.21 b	31.2 ± 2.33 b
Synthetic fungicides	167.7 ± 3.14 a	93.8 ± 2.88 a
CV (%)	35.74	40.13

^y^ *Trichoderma* spp.: *T. azevedoi*, *T. afroharzianum*, *T. asperellum* and *T. asperelloides*. ^z^ Synthetic fungicides: Mancozeb (5 L ha^−1^), fluazinam (0.5 L ha^−1^) and tolclofos-methyl (3 L ha^−1^). * Means of nine replications with a common letter within a column are not significantly different according to Tukey’s test (*p* = 0.05).

**Table 5:** Effectiveness of a combination of four *Trichoderma* species, synthetic fungicides and their alternate application on the incidence and severity of potato tuber white mold under field conditions.

Treatment	Experiment 2021		Experiment 2022	
	Incidence (%)	Severity (%) ^w^	Incidence (%)	Severity (%) ^w^
*Trichoderma* spp. ^x^	1.6 ± 4.75 b *	0.4 ± 2.55 b *	1.3 ± 0.87 b ^z^	0.3 ± 1.12 b *
*Trichoderma* spp. alternating with synthetic fungicides ^y^	4.5 ± 6.22 ab	1.0 ± 2.84 ab	3.3 ± 2.77 ab	0.7 ± 2.46 ab
Synthetic fungicides	7.4 ± 7.78 a	1.5 ± 3.14 a	6.2 ± 4.42 a	1.3 ± 2.27 a
CV (%)	60.64	56.99	57.0	50.94

^w^ Disease severity was determined using the formula by Townsend and Heuberger [31]. ^x^ *Trichoderma* spp.: *T. azevedoi*, *T. afroharzianum*, *T. asperellum* and *T. asperelloides*. ^y^ Synthetic fungicides: Mancozeb (5 L ha^−1^), fluazinam (0.5 L ha^−1^) and tolclofos-methyl (3 L ha^−1^). ^z^ The data were analyzed using the nonparametric Kruskal–Wallis (*p* ≤ 0.05), statistical procedure, and mean separation was performed using the Conover procedure. * Means of nine replications with a common letter within a column are not significantly different according to Tukey’s test (*p* = 0.05).

**Table 6:** Effect of four *Trichoderma* species, synthetic fungicides and their alternate application on potato yield under field conditions.

Treatment	Experiment 2021	Experiment 2022
	t ha^−1^	t ha^−1^
*Trichoderma* spp. ^y^	46.0 ± 3.13 a *	52.9 ± 5.04 a
*Trichoderma* + Synthetic Fungicides	44.1 ± 2.56 ab	48.3 ± 5.45 ab
Synthetic fungicides ^z^	42.4 ± 2.15 b	44.5 ± 7.87 b
CV (%)	5.98	12.87

^y^ *Trichoderma* spp.: *T. azevedoi*, *T. afroharzianum*, *T. asperellum* and *T. asperelloides*. ^z^ Synthetic fungicides: Mancozeb (5 L ha^−1^), fluazinam (0.5 L ha^−1^) and tolclofos-methyl (3 L ha^−1^). * Means of nine replications with a common letter within a column are not significantly different according to Tukey’s test (*p* = 0.05).

**Table 7:** GenBank accession numbers of *Sclerotinia sclerotiorum* and *Trichoderma* spp. isolates used in this study.

Species/Isolate	Locality/Georeference	Year of Collection	Code in Gen Bank
*S*. *sclerotiorum/*SS1	Ahome, Sinaloa/25.819501 -108.955445	2021	PX471991.1
*T. asperelloides/*TES24	Caborca, Sonora/31.06666 -112.338333	2020	OR521164
*T. azevedoi/*TAI73	Ahome, Sinaloa/25.818885 -108.956014	2021	OR521181
*T. afroharzianum/*TAF75	Ahome, Sinaloa/25.491445 -108.571659	2021	OR521183
*T. asperellum/*TAM74	Ahome, Sinaloa/25.491445 -108.571659	2021	OR521182

## Data Availability

The original contributions presented in this study are included in the article. Further inquiries can be directed to the corresponding author.
